# PnPP-15, a Synthetic Peptide Derived from a Toxin from *Phoneutria nigriventer* Spider Venom, Alleviates Diabetic Neuropathic Pain and Acts Synergistically with Pregabalin in Mice

**DOI:** 10.3390/toxins15090560

**Published:** 2023-09-07

**Authors:** Xavier Maia Mariano, Luana Caroline de Assis Ferreira, Camila Megale Almeida-Leite, Célio José de Castro Junior, Maria Elena de Lima

**Affiliations:** 1 Programa de Pós Graduação em Medicina e Biomedicina da Faculdade Santa Casa de Belo Horizonte, Belo Horizonte 30150-240, MG, Brazil; xaviermariano@faculdadesantacasabh.edu.br (X.M.M.); luana.assisferreira@gmail.com (L.C.d.A.F.); celiojunior@faculdadesantacasabh.edu.br (C.J.d.C.J.); 2 Departamento de Morfologia, Instituto de Ciências Biológicas, Universidade Federal de Minas Gerais, Belo Horizonte 31270-901, MG, Brazil; camila@icb.ufmg.br

**Keywords:** PnPP-15 peptide, *Phoneutria nigriventer* spider, neuropatic pain in diabetes, pregabalin, antinociception

## Abstract

Diabetic neuropathic pain is one of the complications that affect a wide variety of the diabetic population and is often difficult to treat. Only a small number of patients experience pain relief, which usually comes with onerous side effects and low levels of satisfaction. The search for new analgesic drugs is necessary, given the limitations that current drugs present. Combining drugs to treat neuropathic pain has been attracting interest to improve their efficacy compared to single-drug monotherapies while also reducing dose sizes to minimize side effects. The aim of our study was to verify the antinociceptive effect of a synthetic peptide, PnPP-15, alone and combined with pregabalin, in male Swiss diabetic mice using the von Frey method. PnPP-15 is a synthetic peptide derived from PnPP19, a peptide representing a discontinuous epitope of the primary structure of the toxin PnTx2-6 from the venom of the spider *Phoneutria nigriventer*. The antinociceptive activity of both compounds was dose-dependent and showed synergism, which was verified by isobolographic analysis. Treatment with PnPP-15 did not cause spontaneous or forced motor changes and did not cause any damage or signs of toxicity in the analyzed organs (pancreas, lung, heart, kidney, brain, or liver). In conclusion, PnPP-15 is a great candidate for an analgesic drug against neuropathic pain caused by diabetes and exerts a synergistic effect when combined with pregabalin, allowing for even more efficient treatment.

## 1. Introduction

The prevalence of diabetes mellitus (DM) and its chronic complications continue to increase at an alarming rate [1]. The International Diabetes Federation (IDF) has estimated that 537 million people worldwide are diabetic [2]. Peripheral neuropathy is a frequent complication of chronic diabetes that most commonly presents as a distal degenerative polyneuropathy with sensory loss [3,4,5]. Around 20–50% of such patients may also experience neuropathic pain, and this condition is recognized as one of the most difficult pain syndromes to treat, and the results are often unsatisfactory [6]. There are currently few treatments that alleviate diabetes-induced neuropathy, but their use is limited due to the side effects of most agents [6,7,8]. Thus, it is necessary to develop new drugs for the effective treatment of neuropathic pain related to diabetes. In this context, animal-derived toxins have been revealed in several active substances with prospective therapeutic applications, including peptides with antinociceptive activity.

Toxins and peptides derived from the venom of the spider *Phoneutria nigriventer* were tested in pain models and demonstrated antinociceptive effects [9,10,11,12,13,14,15,16,17,18]. The peptide used in our study, called PnPP-15, is derived from PnPP-19, which is a synthetic peptide first obtained in silico, having the toxin PnTx2-6 of the spider *P. nigriventer* as a template. The PnPP-15 peptide was found in studies with PnPP-19, which, when topically applied to human skin (ex vivo), was permeabilized by it but suffered proteolysis by an enzyme of that tissue, losing four amino acid residues, generating the PnPP-15 [19].

PnPP-19 induced peripheral [17] and central [18] antinociception in rats in models of inflammatory pain involving the opioid and cannabinoid systems. Furthermore, it was verified through histopathological experiments that PnPP-19 did not induce any signs of toxicity in different tissues (brain, heart, lung, liver, and kidney) and did not cause death or significant immunogenicity in mice, even at high doses [20]. In another study [21], it was shown that PnPP-19 acts with specificity on µ-type opioid receptors, indirectly modulating calcium currents in DRG neurons.

Combined therapies for the treatment of neuropathic pain have been attracting interest with the goal of improving efficacy in relation to drugs administered as monotherapies, also allowing reduced doses to minimize side effects [22,23]. Our study has the general objective of verifying the antinociceptive effect of the synthetic peptide, PnPP-15, derived from a toxin from the venom of the spider *Phoneutria nigriventer*, and when the same peptide is combined with pregabalin, in male Swiss diabetic mice. The von Frey method was used to assess nociceptive activity.

The experimental hypothesis is that the peptide derived from PnPP19, i.e., PnPP-15, also has an antinociceptive effect. In addition, its effectiveness has been tested in combination with pregabalin, a drug used as an analgesic, including for pain in diabetes. Considering the antinociceptive effect of PnPP-15 on diabetic neuropathic pain, we evaluated the effects of the isolated compound on motor function in Swiss *naive* mice. We evaluated spontaneous and forced locomotor activity through open field and rotarod tests, respectively. Moreover, histopathological analysis (HE) of tissues was performed to verify possible injury/toxicity by the studied peptide, as well as the streptozotocin-induced diabetic model.

## 2. Results

### 2.1. PnPP-15 Alone or Combined with Pregabalin Was Effective in Reversing Streptozotocin-Induced Mechanical Allodynia

**Figure 1:**
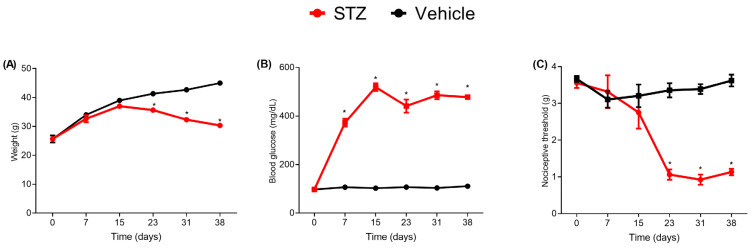
Characterization of the pain model related to diabetic neuropathy. Effect of streptozotocin (STZ, 90 mg/kg i.p.) or vehicle (Citrate) on weight (g) (**A**), blood glucose levels (**B**), and nociceptive threshold by von Frey filaments (**C**), (*n* = 6) per group. Values are expressed as the mean ± standard error of the mean (X ± SEM) measured on days 0, 7, 15, 23, 31, and 38. Student’s *t*-test for unpaired data, * *p* < 0.05.

A single dose of streptozotocin (90 mg/kg, I.P.) leads to a marked increase in blood glucose levels that reaches statistical significance from the 7th day and stands up to the last analyzed day (38th day, Figure 1B). A slight weight loss was seen in streptozotocin compared to the saline-treated group that reaches statistical significance on the 23rd day post injection (Figure 1A). Alongside the increased glucose levels, the mechanical sensitivity in the streptozotocin group significantly increased compared to the saline group as a sign of tactile allodynia, especially after the 3rd week, as can be seen by the reduction in nociceptive threshold measured by von Frey filaments (Figure 1C).

**Figure 2:**
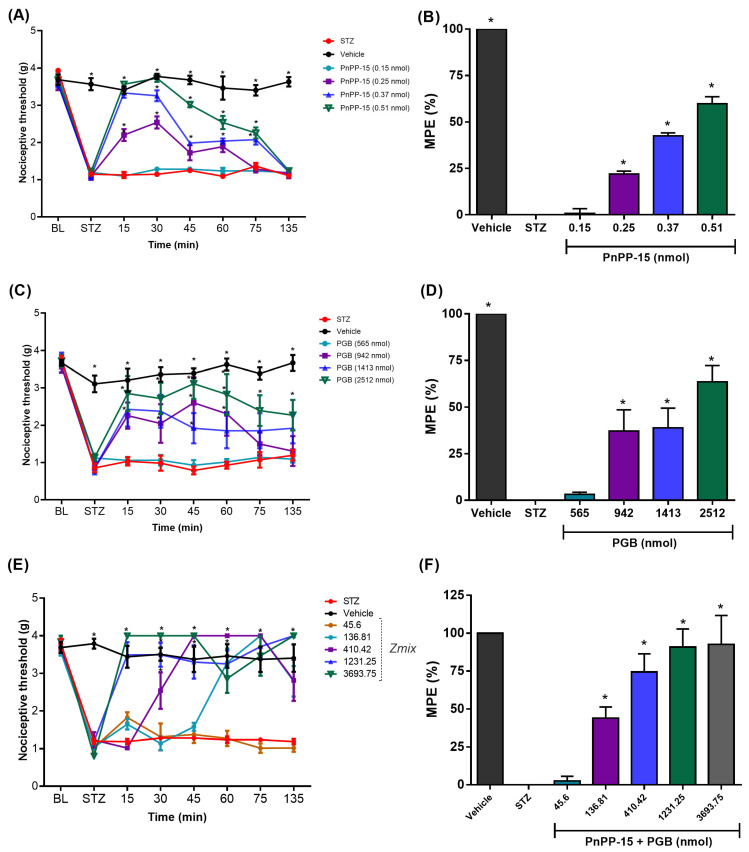
Effect of PnPP-15, PGB, and their combination after intraplantar administration in different doses. The BL (Baseline) shows the threshold before STZ, mechanical allodynia can be seen on the STZ time point. Time and dose-response curve of PnPP-15 (**A**), PGB (**C**), and PnPP-15 + PGB (**E**). Their respective maximum possible effects (MPE) were calculated through the area under the curve, for 135 min, PnPP-15 (**B**), PGB (**D**), and PnPP-15 + PGB (**F**). Measurements began fifteen minutes after peptide administration and were performed every fifteen minutes. Time and dose-response curves were analyzed using two-way repeated measures ANOVA followed by Post hoc Bonferroni test. The maximum possible effect (MPE%) plot was analyzed using one-way ANOVA followed by Dunnett’s Post hoc test. Data are expressed as mean ± SEM, (*n* = 6 animals per group). * *p* < 0.05 concerning the STZ group.

The intraplantar treatment with PnPP-15 peptide showed a dose-dependent antinociceptive effect on tactile allodynia (Figure 2A). Doses of 0.25, 0.37, and 0.51 nmol of the peptide injected into the posterior hind paw of male Swiss diabetic mice were able to reverse the allodynia. The dose of 0.51 nmol reached the reversal of allodynia in diabetic animals 30 min after its administration, with its antinociceptive effect lasting up to 75 min when compared to untreated diabetic animals. The dose of 0.51 nmol of PnPP-15 (Figure 2B) reversed the maximum possible effect by 59.85 ± 3.73%, calculated by the area under the curve (AUC) for the 135 min of the experiment and was statistically significant when compared to diabetic animals, which did not receive the treatment, by the one-way ANOVA test, followed by the post hoc Dunnett test (*p* < 0.001). The curves for the 0.25 nmol and 0.37 nmol dose also showed an antinociceptive effect 15 min after administration and lasted up to 60 and 75 min with a reversal of allodynia at 21.98 ± 1.57 and 42.49 ± 1.65 percent, respectively. The doses of 942, 1413, and 2512 nmol of PGB administered intraplantarly in the right hind paw of animals induced with diabetes also showed a significant antinociceptive effect, attenuating tactile allodynia in a dose-dependent manner (Figure 2C). The dose of 2512 nmol of PGB showed an antinociceptive effect (*p* < 0.05) after 15 min, with the maximum peak of action reached at 45 min after its administration. The evaluation of the area under the curve during the 135 min of the experiment (Figure 2D) showed that PGB (2512 nmol) reversed in 63.37 ± 7.35% of the STZ-induced allodynia. The antinociceptive effects of doses of 942 nmol and 1413 nmol of PGB were seen after 15 min of drug administration (*p* < 0.05) and lasted up to 60 min (*p* < 0.05). These doses (942 nmol) of PGB (Figure 2D) showed a reversal of 37.20 ± 9.62 and 38.93 ± 8.91 percent of the maximum possible effect (%MPE) calculated using the area under the curve (AUC) for the duration of 135 min of the experiment and was statistically significant when compared to diabetic animals that did not receive the treatment (one-way ANOVA test, followed by the post hoc Dunnett test *p* < 0.001).

### 2.2. Synergistic Interaction between PnPP-15 and Pregabalin

**Figure 3:**
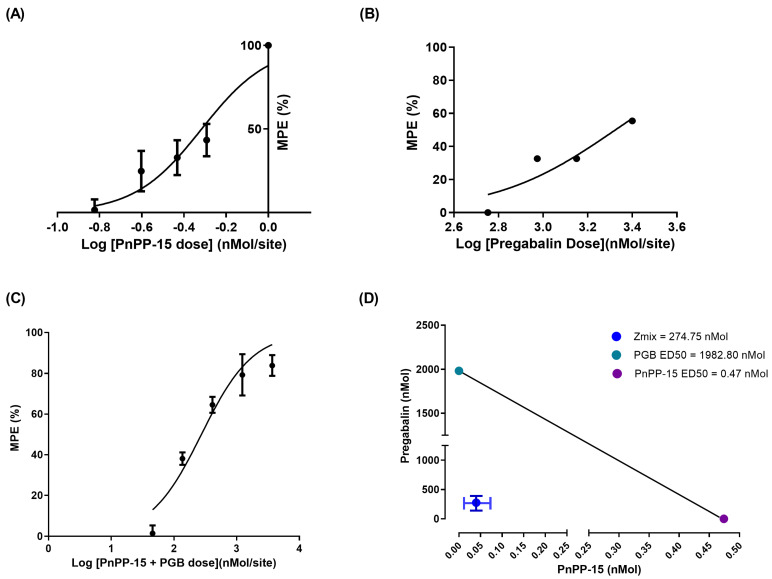
Combination of PnPP-15 and PGB plotted on an isobologram. Dose-response curve of PnPP-15 (**A**), PGB (**B**), and their combination (**C**). The Isobologram (**D**) PGB ED_50_ (y-axis) vs. PnPP-15 (x-axis) with the theoretical additive line connecting the ED_50_ values. The point inside the graph represents the ED_50_ experimentally obtained ED_50_, Z_mix_ (with 95% confidence intervals) indicating a synergistic interaction.

The evaluation of the intraplantar administration of PnPP-15 and pregabalin in the right hind paw of the animals using the von Frey behavioral test in experimental diabetic neuropathic pain induced by STZ showed that the peptide, or pregabalin, produced a dose-dependent antinociceptive effect, as shown in Figure 2A and Figure 2C, respectively. PnPP-15 showed a potency (ratio between pregabalin ED_50_ and PnPP-15 ED_50_) 4218 times greater than PGB, as shown in Figure 3A,B. After estimating the variances of the ED_50_ values from the effect of drugs given alone, the calculated “f” was 0.38 and the dosage of the two components on each drug pair is presented at Table 1. The experimentally obtained ED_50_ value (Z_mix_) with 95% confidence intervals of PnPP-15 combined with PGB was 274.5 (261.67–374.32) nmol/site, (Table 2). The combination of drugs also produced a dose-dependent effect on antinociception (Figure 2E,F and Table 1, *p* < 0.05). The combination doses 136.81, 410.42, 1231.25, and 3693.75 nmol of PnPP-15 and PGB reversed dose-dependent allodynia. The dose–response curve showed that the joint dose of 136.81 nmol of PnPP-15 and PGB had an antinociceptive effect from 60 min and lasted up to 135 min defined by the experiment (*p* < 0.05). In the case of combination doses of 410.42, 1231.25, and 3693.75 nmol of PnPP-15 and PGB, the antinociceptive effects were seen from 30 min after administration of the joint doses and lasted until 135 min defined by the experiment (*p* < 0.05). The combination doses 136.81, 410.42, 1231.25, and 3693.75 nmol of PnPP-15 and PGB reached the same maximum peak of allodynia reversal, although not always at the same times. AUC data normalized (Figure 2F) from doses 136.81, 410.42, 1231.25, and 3693.75 nmol of 135 min after administration of joint doses of PnPP-15 and PGB reverted to 43.94 ± 7.36, 74.35 ± 12.02, 90.85 ± 11.93, and 92.65 ± 19.07 percent the allodynia of the diabetic animals, respectively. The interaction was synergistic with theoretical ED_50_ values (95% CI) of the combination of PnPP-15 with pregabalin was 1231.25 nmol (945.43–1603.47) at 135 min of experiment duration (3C). The experimental ED_50_ (95% C.I) obtained was 274.75 nmol (201.67–374.32) during 135 min of the experiment. The interaction index (I.I.) was 0.22. The isobologram is shown in Figure 3D.

### 2.3. PnPP-15 Antiallodynic Effect Is Not Accompanied by Side Effects

**Figure 4:**
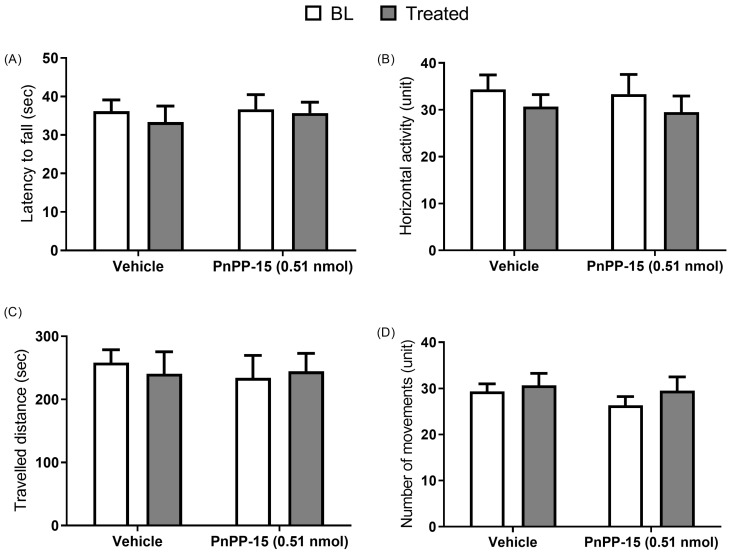
Forced and spontaneous motor performance in mice is not affected by PnPP-15. Measurements were taken before (gray bars) and 30 min after treatment (white bars). For the rotarod test, training was given (three days before for learning warranty. The latency of the animal to fall was measured on the 4th day (**A**). For spontaneous performance, data were recorded over 5 min, and tests evaluated horizontal activity (**B**), total distance (**C**), and total number of movements (**D**). Results are expressed as median ± SEM (*n* = 6 animals per group). No statistical difference was observed when analyzed using the Wilcoxon non-parametric test.

**Figure 5:**
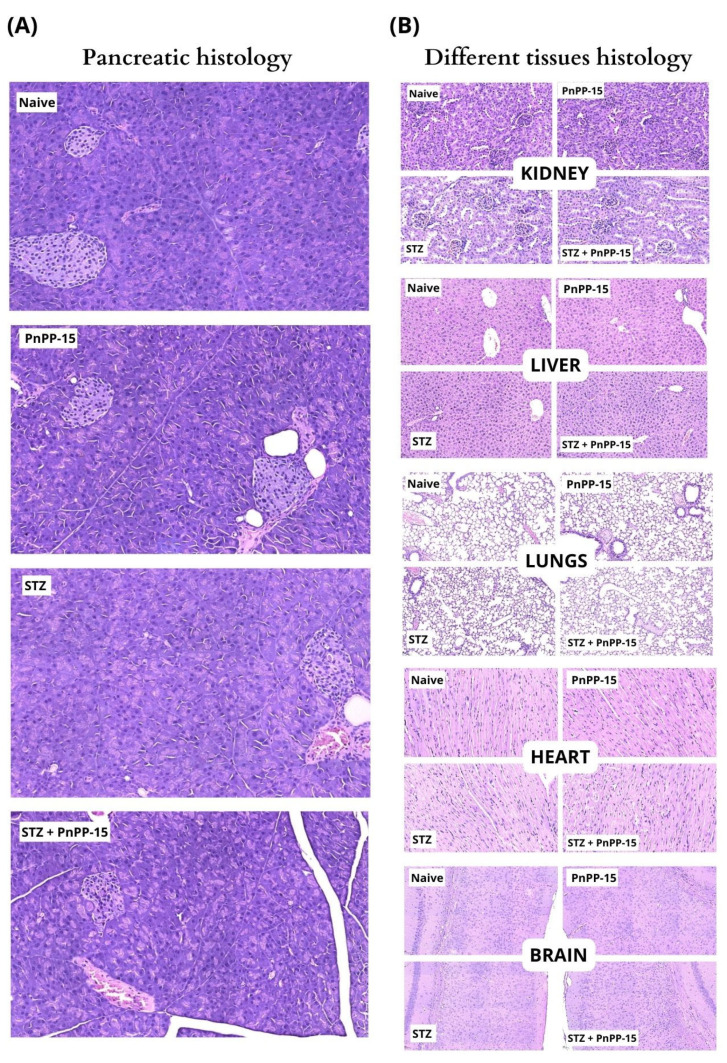
The injection of the peptide (5 mg/kg) did not cause any damage or signs of toxicity in the analyzed organs: (**A**) pancreas or (**B**) kidney, liver, lung, heart and brain. (**A**) Pancreas showed preserved histology in naïve or PnPP-15 mice. STZ and STZ + PnPP-15 diabetic mice showed pancreas with lower number of Langerhans islets and insulinitis. (**B**) Kidney, liver, lung, heart and brain showed preserved histology in all groups.

The assessment of adverse effects was performed without any specific pain model; *naïve* Swiss animals were used for open field, rotarod, and as a control for histological analysis. The peptide was administered at a dose of 0.51 nmol, and the injection was performed 25 min before behavioral tests (Figure 4B–D). PnPP-15 did not compromise the forced or spontaneous locomotion of the animals (Figure 4A–D). Histological analysis (Figure 5) showed that diabetic mice had changes in the pancreas, with fewer islets of Langerhans and insulitis compared to non-diabetic animals. However, the peptide injection did not cause any damage or signs of toxicity in the other analyzed organs (pancreas, lung, heart, kidney, brain, or liver).

The BL (Baseline) shows the threshold before STZ, and mechanical allodynia can be seen on the STZ time point. Time and dose–response curve of PnPP-15 (2A), PGB (2C), and PnPP-15 + PGB (2E). Their respective maximum possible effects (MPE) were calculated through the area under the curve for 135 min (PnPP-15 (2B), PGB (2D), and PnPP-15 + PGB (2F)). Measurements began fifteen minutes after peptide administration and were performed every fifteen minutes. Time and dose–response curves were analyzed using two-way repeated measures ANOVA followed by the post hoc Bonferroni test. The maximum possible effect (MPE%) plot was analyzed using one-way ANOVA followed by Dunnett’s post hoc test. Data are expressed as mean ± SEM, (*n* = 6 animals per group). * *p* < 0.05 concerning the STZ group.

Dose–response curve of PnPP-15 (3A), PGB (3B), and their combination (3C). The isobologram (3D) PGB ED_50_ (y-axis) vs. PnPP-15 (x-axis) with the theoretical additive line connecting the ED_50_ values. The point inside the graph represents the ED_50_ experimentally obtained ED_50_, Z_mix_ (with 95% confidence intervals), indicating a synergistic interaction.

The pancreas showed preserved histology with islets of Langerhans (arrow) surrounded by exocrine cells in control mice (naive) and control mice injected with PnPP-15. Diabetic mice (STZ) had a pancreas with a lower number of islets of Langerhans and insulins, which consisted of islets with greater cellularity due to the infiltration of mononuclear inflammatory cells. The pancreas of diabetic mice injected with peptide (STZ + PnPP-15) showed a similar appearance compared to diabetic mice. 

## 3. Discussion

The present study showed that the synthetic peptide PnPP-15 causes antinociception in a dose-dependent manner in male Swiss mice suffering from STZ-induced diabetes. Moreover, the concomitant administration of PnPP-15 and pregabalin produced analgesic potentiation that was greater than simple additivity, which is synergic. Previous data with PnPP19, which is the precursor of PnPP15, revealed that the mechanism of this molecule involves dose-dependent activation of µ-opioid receptors and CB1 cannabinoid receptors [17]. Preliminary (unpublished) data show that this mechanism is also observed for PnPP15. The activation of µ-opioid and CB1 cannabinoid receptors with the selective agonist PnPP-15 may explain the antinociception observed in animals with diabetic neuropathic pain in the present study. This finding reinforces the possibility that opioid and cannabinoid receptors work together within the same cell or neuronal circuit to produce antinociception and that modulation of one receptor system can lead to changes in the activity of the other [25].

One of the neuropathic pain treatment strategies involves the joint use of opioid and cannabinoid agonists [26,27,28,29,30]. One could choose to combine PnPP-15, a peptide that possibly acts on the opioid and cannabinoid system, with an opioid or cannabinoid agonist already used clinically. However, interactions between opioid and cannabinoid receptors, in addition to mediating only the production of acute antinociception, can supply undesirable side effects, such as the development of tolerance and reward. The other strategy used for the treatment of neuropathic pain involves drugs that affect multiple processes rather than a single specific target, positioning itself as the greatest promise for future therapeutic development [30]. Based on this premise, we understand that combined therapy for the treatment of diabetic neuropathic pain, with a synergistic effect, using two drugs with the possibility of different mechanisms of action, could be a useful and highly effective strategy, hence the interest in combining the PnPP-15 peptide with pregabalin.

PnPP-15, which, as already mentioned, seems to act via the opioid and cannabinoid systems, similar to its precursor peptide PnPP-19 (Freitas et al., 2016) [17]. However, PnPP-15 is a shorter peptide than PnPP-19, which can be positive concerning production cost, among others. In addition, PnPP-19 has never been tested combined with other analgesic drugs, although different preclinic tests were already carried out with this peptide, considering its possible use to treat erectile dysfunction, supporting that it seems to be safe as a pharmaceutical drug. Considering the similar effects, near structure, and action mechanism of PnPP-15 compared to PnPP-19, in addressing pain, we may suppose that PnPP-15 may also be safe. Indeed, it may be confirmed. On the other hand, pregabalin is a drug currently in use and considered first-line for the treatment of diabetic neuropathic pain, acting on the α2δ-1 subunit of calcium channels [6]. There are also reports of satisfactory clinical use of combinations of morphine and pregabalin, which reinforces the idea of a beneficial multimodal combination of drugs involving the opioid system and the α2δ-1 subunit of voltage-gated calcium channels (VGCCs) [31]. As PnPP-15 and PGB can affect different targets, all involved in pain modulation, this could explain the synergism. This strategy opens the prospect of reducing the development of tolerance and dependence, euphoric effects, and withdrawal when high doses of pregabalin are administered and abruptly stopped [32].

Moreover, there are still no studies about possible dependence or tolerance due to prolonged use of the PnPP-15 peptide, considering its action on the opioid receptor. However, further studies on the mechanisms of action of PnPP-15 and its combination with pregabalin are needed to understand its effects and synergy in the antinociception process. On the one hand, pregabalin and PnPP-15 reversed tactile sensitivity in diabetic mice; on the other hand, PnPP-15 showed a potency 4218 times greater than pregabalin (ratio between ED_50_ of pregabalin and ED_50_ of PnPP-15), requiring lower doses of PnPP-15 to produce antinociception, which reduces the possibility of significant side effects occurring compared to pregabalin, as shown in Figure 3B and Figure 3A, respectively. In view of the antinociceptive action of PnPP-15 in diabetic neuropathic pain and considering its potential use as a drug, we evaluated its possible effects on motor function in Swiss naive mice (without diabetes), as well as possible damage to different tissues through histological analysis. Spontaneous and forced locomotor activity was evaluated in mice submitted to open field and rotarod tests, respectively. PnPP-15 administered at a dose of 0.51 nmol did not compromise the forced or spontaneous locomotion of the animals, as evaluated in the rotarod test and in the open field (Figure 4A–D). On the other hand, the histological analysis (Figure 5) showed that, as already proven [33], diabetic mice presented histological alterations in the pancreas, with a smaller number of islets of Langerhans and with insulitis, in comparison to non-diabetic animals. However, the injection of 5 mg/kg of PnPP-15 diluted in saline by the intraperitoneal route did not cause any damage or signs of toxicity in the analyzed organs (pancreas, lung, heart, kidney, brain, or liver). These results corroborate the histopathological studies already conducted with PnPP-19 [20], the precursor peptide of PnPP-15, in Wistar rats. In that study, none of the tissues analyzed (kidney, heart, liver, lung, and brain) showed signs of toxicity by the PnPP-19 peptide at a dose of 5 mg/kg (peptide dose/animal weight), administered intraperitoneally.

Therefore, from the results obtained in the motor tests in mice and the histological analysis of the tissues of the animals treated with PnPP-15, no clear sign of toxicity of this peptide was verified in the referred animals. However, it is known that any drug candidate must undergo a series of pre-clinical tests, and the analysis conducted here with PnPP-15 is a positive indication that it is not toxic, but other tests must be conducted to assert a safe conclusion from that parameter.

## 4. Conclusions

It was shown that PnPP-15 is a workable candidate for an analgesic drug against neuropathic pain caused by diabetes and that, when administered with pregabalin exerts a synergistic effect. This is relevant because lower doses of pregabalin can be administered with greater efficiency when combined with the peptide, and possibly, avoiding side effects. However, it must be considered that the route of administration of the peptide (injectable) is not the best accepted in the case of a treatment, but future studies may contribute to enabling other possible routes of administration, perhaps involving some formulation of this peptide. A positive point is the fact that the peptide did not show toxicity, at least considering the motor tests and histological analyses.

## 5. Materials and Methods

### 5.1. Experimental Animals

The project was approved under protocol number 001/2020 by the Ethics Committee for Research Involving Animal Experimentation of Santa Casa de Belo Horizonte (CEPEEA). The experiments reported in this study were performed in accordance with the National Institutes of Health Guide for the Care and Use of Laboratory Animals (NIH publications No. 8023, revised 1978) [34] and 33 in accordance with the Brazilian Guidelines for the Care and Use of Animals in Scientific Research—DBCA (Normative No. 30, 2016) [35]. Swiss mice (*Mus musculus*), aged between 4 and 6 weeks and male, were provided by the Experimental Animal Center of the Federal University of Minas Gerais (CEBIO/UFMG). The induction of diabetes began when they reached a weight of 20 to 25 g. All animals were kept in polypropylene cages and kept under a cycle of 12 h of light and 12 h of dark. Standard food and water were supplied *ad libitum*.

### 5.2. Drugs

PnPP-15 (Synthesized by Genone, Rio de Janeiro, Brazil) was diluted in physiological saline. Pregabalin (Pregabalin, brand LGC Ltd., Luckenwalde, Germany). Streptozotocin (Sigma Co., San Luis, TX, USA).

### 5.3. Mechanical Allodynia

The animals were placed in cages with a wire mesh bottom for 20 min to adapt to the unfamiliar environment. A series of von Frey filaments (0.07–4 g) were applied perpendicularly to the surface region of the right hind paw to the extent that it caused a bending of the filament. Each von Frey filament is applied for a period of up to 2 s (cutting time) or until the animal demonstrates an escape response (paw withdraw threshold—PWT). However, in the absence of a positive response, the next stronger von Frey filament is applied. This procedure is repeated up to four measurements after a change in the first response or up to five consecutive negative responses. A force of 4 g was selected as the cutting force at which the application of the filaments is stopped, adapted from [36]. Before drug administration, selected diabetic animals were evaluated to obtain the baseline nociceptive threshold. The antiallodynic action of the peptides and pregabalin was evaluated 15, 30, 45, 60, 75, and 135 min after the administration of these compounds. The doses of PnPP-15 and pregabalin evaluated for the construction of the curve were 0.15, 0.25, 0.37, and 0.51 nmol and 565, 942, 1413, and 2512 nmol, respectively. All animals were administered with a volume of 10 µL of saline or saline containing the drugs.

### 5.4. Diabetes Induction

A total of 90 mg/kg of STZ diluted in sodium citrate buffer 0.1 M was given i.p (intraperitoneal) as a single dose to the mice, and the regular water was replaced by glucose water at 10%. At the end of this protocol, the establishment of type I diabetes was confirmed with blood glucose measured using the Accu-Chek Active System (Roche Diagnostics) device. Animals with glycemia equal to or greater than 300 mg/dL were considered diabetic and included in the experimental group. Weight and glycemia were checked seven days after the induction of diabetes until the twenty-eighth day. A preliminary study conducted by us, lasting 38 days, showed that the maximum level of tactile sensitivity in animals induced to diabetes was reached from the 23rd day onwards day when the weight also began to decrease. It was decided to set a time of 28 days to start the nociception tests. The mice in the control group were injected with saline solution in a volume corresponding to the other tests.

### 5.5. Drug Combination and Isobolographic Analysis

Least squares linear regression analysis of the log dose–response curves for PnPP-15 and PGB supplied the calculation of the dose that produced 50% of antinociception [37,38]. PnPP-15 and PGB dose pairs were derived from the ED_50_ values of the individual drugs. They were co-administered 15 min before the von Frey paw withdrawal test (PWT), as previously described [36] in six animals per dose, by intraplantar injection route. The isobologram was constructed with the values derived from the ED_50_ of PnPP-15 on the abscissa and the values derived from the ED_50_ of pregabalin on the ordinate, generating the additivity line and the correspondent theoretical ED_50_ for an additive interaction (here called Z_add_). The experimental ED_50_ of the mixture was obtained by linear regression analysis of the dose–response curve, considering the corresponding logarithmic dose of the mixture, and compared with the Z_add_ value by the *t*-test.

The theoretical Z_add_ was deduced from the formula below:

$$Z_{add}=f\times \left[PnPP\text{-}15\right]+\left(1-f\right)\times [PGB]$$

where the f value was obtained through the equation

$$f=\frac{{VED}_{50}\;of\;PGB}{{VED}_{50}\;of\;PGB+{VED}_{50}\;of\;PnPP\text{-}15}$$

and VED_50_ corresponds to the variance value of the dose of PGB or PnPP-15 that causes 50% of the maximum effect possible (%MPE). This f value was used to calculate the additivity and combination ratio of PnPP-15 and PGB according to the equations:

$${\left(PnPP\text{-}15\right)}_{add}=f\times {ED}_{50}\;of\;PnPP\text{-}15\;and\;{\left(PGB\right)}_{add}=\left(1-f\right)\times {ED}_{50}\;of\;PGB$$

The sum of these is the Additivity value called by Z_add_ for the selected effect level (50%) and is the expected value to give the 50% effect. The point is that the experimental ED_50_ is located on the isobologram, and the location on the graph where the experimental point is located determines the type of interaction. If the experimental point is below the additivity line and is statistically different from the additivity point, the effect of combining PnPP-15 with PGB will be synergistic or super additive. To certify the nature of the drug mixture, the interaction index (I.I.) is also calculated with the following formula: I.I. = experimental ED_50_/theoretical ED_50_. I.I. < 1 indicates synergism, while an I.I. > 1 indicates antagonism and additivity when I.I. = 1.

### 5.6. Motor Activity Measurements

Motor function was assessed by the spontaneous activity that the animal performed. An open-field apparatus consisting of a box measuring 25 cm × 25 cm with a floor divided into nine areas was used. The animal was placed in it to perform free and uninterrupted movements along the quadrant of the maze. The movements performed by the mice were recorded by software, including the duration and number of these movements as well as the total distance covered during 300 s after 25 min previously administered the drug by intraplantar injection. The median of the tests stands for each parameter for each animal.

For the rotarod test, the animal was placed in a rotating cylinder at an acceleration rate of 4 rpm to 40 rpm during the first 120 s and a total rotation time of 300 s, forcing the animals to move to maintain balance. A priori, the animals performed three acclimatization runs for three consecutive days. Only naive animals were used to exclude the possibility of biases related to motor function deficits associated with diabetes [37,39]. The median of the 3 tests represents each parameter for each animal. Behavioral open field and forced locomotion tests were performed in a quiet room between 1 pm and 3 pm.

### 5.7. Histopathological Analysis (HE)

Non-diabetic control (nD) mice were injected with saline, diabetic control (D) mice were injected with saline, non-diabetic animals were injected with the peptide (nDp), and diabetic mice were injected with the peptide (Dp); 2 per group were sacrificed by cervical dislocation 2 h after intraperitoneal injection of 5 mg/kg of PnPP-15 diluted in saline. Brain, heart, lung, kidney, pancreas, and liver were isolated, sliced, and immersed in 4% paraformaldehyde buffered solution for further embedding in paraffin and routine staining with hematoxylin and eosin (HE). The microscopic slides were digitized by a Pannoramic MIDI scanner (3D Histech, Budapest, Hungary) at the Image Acquisition and Processing Center (CAPI) of the Biological Institute from the Federal University of Minas Gerais. Tissues were analyzed by the pathologist, and digital images were acquired for documentation using CaseViewer 1.4 software (3D Histech, Budapest, Hungary).

### 5.8. Data Analysis

Results are expressed as mean ± SEM except for spontaneous and forced locomotion test results, which are presented as median ± range. Statistical analyses were performed using GraphPrism 5 software. Statistical significance was set at *p* < 0.05. All results, except for the histopathological analysis, were given to statistical normality analysis using the Kolmogorov–Smirnov normality test for *p* < 0.05. The results of the dose–response curves were also calculated as area under the curve (AUC) and normalized to the percentage of the maximum possible effect (%MPE) of the 135 min of duration of the experiment. For the induction of type I diabetes and mechanical allodynia, the Student’s *t*-test was used to compare two independent groups. Dose–response curves were analyzed using two-way repeated measures ANOVA, followed by the post hoc Bonferroni test. Data were calculated as area under the curve (AUC) and normalized to the percentage of maximum possible effect (%MPE) and were analyzed using one-way ANOVA followed by Dunnett’s post hoc test to compare measures of effects with diabetic animals that did not receive the treatment. Calculation of the area under the curve (AUC) of the dose–response curve was over the entire duration of the response measurement (135 min). AUC data were normalized to the percentage of maximum possible effect (%MPE) according to the formula

$$\%MPE=\frac{\left(A-B\right)}{\left(C-B\right)}\times 100$$

where A is the paw withdrawal threshold of each animal in the treated group (drugs alone or in combination). B is the paw withdrawal threshold for each animal with allodynia associated with type I diabetes that did not receive any of the study drugs. C is the nociceptive threshold cutoff point for naïve animals, defined here as 4 g. The results of the forced locomotion test (rotarod) and spontaneous locomotion (open field) were analyzed using the Wilcoxon non-parametric test.

## Figures and Tables

**Table 1:** Combined doses of PnPP-15 and pregabalin were used to assess the type of interaction.

Drugs Pair	PnPP-15 (nmol)	PGB (nmol)	PnPP-15 + PGB (nmol)
1	0.006658475	45.59514913	45.60180760
2	0.019975426	136.7854474	136.8054228
3	0.059926280	410.3563423	410.4162686
4 *	0.179778841	1231.069027	1231.248806
5	0.539336523	3693.207081	3693.746418

Least squares linear regression analysis of the log dose–response curves for PnPP-15 and PGB provided the calculation of the dose that produced 50% antinociception. The PnPP-15 and PGB dose pairs shown in Table 2 were derived from the ED_50_ values of the individual drugs. Line 4 * is a reference to the mixture whose dose refers to the theoretical additive effect of Loewe’s additivity theory [24].

**Table 2:** ED_50_ values of individual and combined drugs for the antinociceptive effect in diabetic mice.

	ED_50_ (PnPP-15) nmol	ED_50_ (Pregabalin) nmol	ED_50_ (Z_add_) nmol	ED_50_ (Z_mix_) nmol
ED_50_ Alone and combined	0.47	1982.81	1231.25	274.75
95% confidence interval	0.31_0.73	1418.05_2772.47	945.43_16.47	201.67_374.32

ED_50_ (50% antinociceptive doses) are expressed in nmol/site. ED_50_ was determined from dose–response curves. The theoretical additive (Z_add_) was calculated based on the dose–response curves of PnPP-15 and pregabalin alone. The combined (Z_mix_) was determined from the experimentally determined dose–response curves of the combination. Values in parentheses are 95% confidence intervals.

## Data Availability

Not applicable.
